# Multicenter retrospective study of biological tolerance in juvenile idiopathic arthritis (jir-cohort)

**DOI:** 10.1186/1546-0096-12-S1-P184

**Published:** 2014-09-17

**Authors:** Natalia Cabrera, Andreas Woerner, Samuel Roethlisberger, Florence Aeschlimann, Carine Wouters, Gerald Berthet, Anuela Kondi, Ettienne Merlin, Daniela Kaiser, Salma Malik, Behrouz Kassai Koupai, Laetitia Higel, Anne Maes, Elvira Cannizzaro, Cyril Jeanneret, Isabelle Kone-Paut, Alexandre Belot, Michael Hofer

**Affiliations:** 1 Rheumatology, Nephrology and Pediatric Dermatology, Hospital Femme Mere Enfant, Lyon , France; 2 Pediatric Rheumatology, University Children's Hospital, Basel, Switzerland; 3 Rhumatologie Pédiatrique Romande, Centre Hospitalier Universitaire Vaudois , Lausanne, France; 4 Pediatric Reumatology, University Children’s Hospital, Zurich, Switzerland; 5 Pediatric Rheumatology, University Hospital, Leuven, Belgium; 6 Pediatrics, Children’s Hospital, Aarau, Switzerland; 7 Pediatrics, Kremlin-Bicêtre Hospital, Paris, France; 8 Pediatrics, Centre Hospitalier Universitaire, Clermont-Ferrand, France; 9 Pediatrics, Children’s Hospital, Lucerne, Switzerland; 10 Centre d'Investigation Clinique de Lyon, EPICIME, Lyon, France; 11 Pediatrics, Hôpital Hautepierre, Strasbourg, France; 12 Pediatric Rheumatology, Kremlin-Bicêtre Hospital, Paris, France

## Introduction

Ten years after the introduction of biologics in children, we assess the tolerance of these therapies among patients with juvenile idiopathic arthritis (JIA) in pediatric rheumatology centers.

## Objectives

Study the epidemiology of JIA in 10 european centers of pediatric rheumatology and describe adverse events (AEs) to biologics at short and medium term.

## Methods

Multicenter retrospective observational study in JIA patients who have been treated with biologics, in the 10 following pediatric rheumatology centers: Basel, Zurich, Aarau, Lucerne, Vaud (Switzerland), Lyon, Paris, Clermont-Ferrand, Strasbourg (France) and Leuven (Belgium) between June 1999 and March 2014.

## Results

531 patients were included in the study. Most of them suffered from polyarticular JIA (PA) RF negative (21.4 %, n = 115) . Other JIA subtypes were distributed as follow: arthritis with enthesitis 19.3% (n= 102), oligoarticular JIA (OA) persistent 17.4% (n= 92), systemic JIA 16.6% (n= 88), OA JIA extent 10.6% (n= 56), psoriatic arthritis and JIA PA RF positive 5.1% (n= 27) and other arthritis 4.5%. Ocular involvement was found in all subtypes of JIA except for polyarticular FR+ consisting of 105 patients of which 19.8% (n=70) with positivity for antinuclear antibodies. Familial history of autoimmunity was identified in more than a quarter of cases (n=146, 27.6 %). A macrophage activation syndrome (MAS) was found in 20 patients, restricted to the systemic JIA subset.

Etanercept was the most common drug and accounted for 47.5 % of all biologics in the cohort. Most of AEs were mild, one third of AEs were due to reversible reactions at the injection (such as rash, fever and pain). Infections represented one third of the AEs and were more frequently secondary to viral agents. Tocilizumab was more often involved in AEs with a frequency of 0.27 AE patients/year, followed by infliximab and canakinumab with 0.17 AE patients/year. No AE was seen with rituximab but this drug represented an overall exposition of 7,7 patients/year. Severe AEs were found in 19 patients (3.4%) including two cases of Hodgkin's disease. The mean exposure duration to biologics at the first occurrence of AEs was 14.4 months. No deaths were reported.

## Conclusion

This multicenter study shows an overall good tolerance of JIA patients to biologics. The main AE are represented by infectious agents and preventive strategies including vaccination should apply. Notably, 2 cases of malignancy, both Hodgkin disease, were identified in the cohort. The prospective follow-up of children treated with biologics, and international efforts, such as Pharmachild project, will improve the quality of data collection and the identification of possible predisposing factors for AE.

## Disclosure of interest

None declared.

